# Structure and Function of Mitochondria-Associated Endoplasmic Reticulum Membranes (MAMs) and Their Role in Cardiovascular Diseases

**DOI:** 10.1155/2021/4578809

**Published:** 2021-07-11

**Authors:** Yi Luan, Ying Luan, Rui-Xia Yuan, Qi Feng, Xing Chen, Yang Yang

**Affiliations:** ^1^Department of Translational Medicine Center, The First Affiliated Hospital of Zhengzhou University, Zhengzhou 450052, China; ^2^Department of Physiology and Neurobiology, School of Basic Medical Sciences, Zhengzhou University, Zhengzhou 450001, China; ^3^Research Institute of Nephrology, Zhengzhou University, Zhengzhou 450052, China

## Abstract

Abnormal function of suborganelles such as mitochondria and endoplasmic reticulum often leads to abnormal function of cardiomyocytes or vascular endothelial cells and cardiovascular disease (CVD). Mitochondria-associated membrane (MAM) is involved in several important cellular functions. Increasing evidence shows that MAM is involved in the pathogenesis of CVD. MAM mediates multiple cellular processes, including calcium homeostasis regulation, lipid metabolism, unfolded protein response, ROS, mitochondrial dynamics, autophagy, apoptosis, and inflammation, which are key risk factors for CVD. In this review, we discuss the structure of MAM and MAM-associated proteins, their role in CVD progression, and the potential use of MAM as the therapeutic targets for CVD treatment.

## 1. Introduction

Cardiovascular disease (CVD) is the leading cause of mortality in humans. Globally, there were nearly 523.2 million new cases and 18.6 million deaths from CVD in 2019. Its incidence has increased by 17.1% over the past decade [1, 2]. In China, CVD is the leading cause of overall death (40% of all disease mortality) [3].

CVD is caused by multiple risk factors and pathological mechanisms [4, 5]. At the cellular level, various aberrations, including metabolic abnormalities, energy deficit, autophagy defect, endoplasmic reticulum (ER) stress, reactive oxygen species (ROS) production, and apoptosis activation, may lead to CVD [6]. A substantial amount of energy, which is mainly produced by mitochondria, is essential for normal physiological function of the heart. Thus, the abnormality (dysfunction or malfunction) of mitochondria is the main cause of these cellular perturbations [7]. Being extremely sensitive to oxidative stress, vascular endothelial cells are easily adapted to changing environments, such as altered oxygen levels, pathogens, and endogenous damaging stimuli [8]. In addition, the circulating immune cells might also affect adjacent vascular endothelial cells (ECs) by the initiation of immune reactions, leading to the earliest stages of atherosclerosis and preceding atherosclerotic plaque formation. Mitochondria are closely involved in these processes, which makes them attractive candidates for therapeutic intervention [9]. Meanwhile, a failing heart is accompanied by defected oxidative phosphorylation (OXPHOS) and decreased ATP levels, contributing to defective cardiac performance [10].

The endoplasmic reticulum (ER), as a multifunctional organelle, provides a distinct subcellular compartment with multiple functions, including lipid biosynthesis, calcium storage, and protein folding and processing [11]. Disturbance in proper ER function may cause ER stress, which in turn causes severe impairment in protein folding and therefore poses the risk of proteotoxicity. ER stress has been highlighted as an important regulator of cardiovascular diseases [12]. For example, CHOP is the most widely studied ER stress biomarker involved in ER stress-associated apoptotic signaling in cardiovascular disease [13]. In atherosclerosis, CHOP is elevated by the unfolded protein response (UPR) in the ER, accompanied with a progression of atherosclerosis in the aorta [14]. Another important protein is the PAK2, a stress-responsive kinase localized in close proximity to the ER membrane. Its depletion in cardiomyocyte leads to defect in ER response, cardiac function, and cardiac cell death after being induced with tunicamycin, thus indicating a protective ER stress response against heart failure through modulation of PAK2 levels [15].

Mitochondria and ER have different living cells' roles. Accumulating evidence suggested an interaction between the two [16]. Mitochondria-associated membranes (MAM), also known as mitochondria-ER contact sites (MERCs), are specialized regions, in which this interaction occurs [17]. Growing evidence shows that MAM has a variety of cellular functions that are vital in the regulation of cardiovascular diseases and can also be potential targets for CVD therapy. In this review, we discussed the structure of MAM and MAM-associated proteins, their role in CVD progression, and the potential use of MAM as the therapeutic targets for CVD treatment.

## 2. The Structural Composition of the Mitochondria-Associated Membranes (MAMs)

The existence of MAMs was first reported in the late 1950s. MAM was then isolated, and its biochemical functions were examined in the 1990s [18]. The first identification related to the composition of MAMs was performed by Gao et al., using limited proteolysis [19], while the first comprehensive analysis of MAM proteome was performed by Zhang et al. [19, 20]. Approximately 991 proteins in the MAM fraction were identified. Later on, Poston et al. identified 1212 candidates, including weak soluble proteins at the MAM [21]. Until now, various proteins (approximately 1000) localized within the MAMs were identified in the brain and liver with the aid of in-depth mass spectrometry analysis [22]. Above all, the components within MAMs were highly conserved among species and tissues. The above proteomic analyses were also conducted under pathological conditions, such as diabetes [23].

Perrone et al. have suggested that MAM is mainly involved in calcium homeostasis, lipid metabolism, and protein transfer between mitochondria and ER [24]. Furthermore, Gao et al. found that MAM is also involved in inflammasome formation, autophagy, ER stress, and mitochondria morphology [25]. The proteins located on MAMs either take part in MAM physical interaction or modulation of the tethering complex in MAMs [26]. Tethering proteins, ranging from Ca^2+^ channels to apoptotic proteins, constitute the molecular bridges combining the ER and mitochondrial membrane together [27]. From a physical point of view, the distance between membranes in MAMs ranges from 10 to 25 nm for smooth ER, and 50 to 80 nm for rough ER. Besides, the coverage of MAMs takes about 4 to 20% of the total mitochondrial surface, depending on its cellular stress and metabolic state [20].

**Figure 1:**
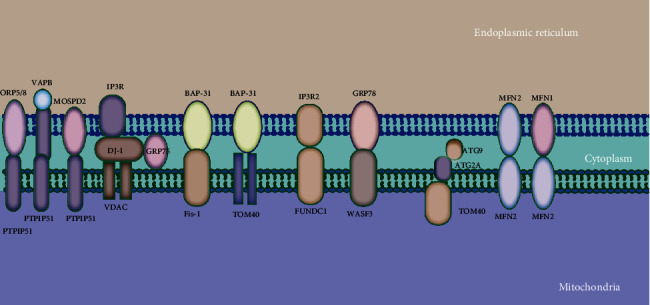
Major mitochondria-ER tethering complexes. Mitochondria are connected to the ER by several protein complexes. The ER protein ORP5/8, VAPB, or MOSPD2 interacts with the mitochondrial PTPIP51. ER-resident IP3R is anchored to OMM-localized protein VDAC via GRP75. The ER-localized BAP31 interacts with the mitochondrial Fis1 and TOM40. The IP3R2 located on the ER partners with the mitochondrial protein FUNDC1. The ER chaperone GRP78 interacts with WASF3. Mitochondrial TOM40 directs ATG2A to MAMs. The ER-localized MFN2 interacts with either MFN1 or MFN2 in the mitochondria.

The well-established MAM proteins include (1) the protethering complexes: (i) mitofusin 2 (MFN2), (ii) the complex formed by vesicle-associated membrane protein-associated protein B (VAPB) and protein tyrosine phosphatase interacting protein 51 (PTPIP51), (iii) PTPIP51 and motile sperm domain-containing protein 2 (MOSPD2), (iv) glucose-regulated protein 75 (GRP75) bridging inositol triphosphate receptor (IP3R) to voltage-dependent anion channel 1 (VDAC1), (v) the mitochondrial fission 1 protein- (Fis1-) B cell receptor-associated protein 31 (BAP31) complex, (vi) the FUN14 domain-containing 1- (FUNDC1-) IP3R2 complex, (vii) the phosphor acidic cluster sorting protein 2 (PACS2), (viii) PDZD8, (ix) Beclin1 (BECN1), and (x) MITOL, Parkin, and AMPK*α*, which regulate MAM formation by directly interacting with MFN2 on the outer mitochondrial membrane (OMM) side; (2) proteins that regulate IP3R/GRP75/VDAC complexes: mitochondrial translocase of the outer membrane 70 (TOM70), Sigma-1 receptor (Sig-1R), cyclophilin D (CypD), pyruvate dehydrogenase kinases 4 (PDK4), thymocyte-expressed, positive selection-associated gene 1 (Tespa1), reticulon 1C (RTN-1C), glycogen synthase kinase-3*β* (GSK3*β*), disrupted-in-schizophrenia 1 (DISC1), transglutaminase type 2 (TGM2), Wolfram syndrome 1 (WFS1), and etoposide-induced protein 2.4 (EI24); (3) antitethering factors: (i) trichoplein/mitostatin (TpMs) that negatively regulates MAMs tethering via MFN2, (ii) FATE1 uncoupling MAMs by interacting with ER chaperones and emerin (EMD) and the mitofilin, (iii) Caveolin-1; and (4) upstream regulators of MAM formation: (i) glycogen synthase kinase-3*β* (GSK3*β*), (ii) p38 MAPK, (iii) cGMP-dependent protein kinase (PKG), (iv) FOXO1, (v) cAMP-dependent protein kinase (PKA), and (vi) AMPK*α* (Figure 1) [28].

According to the localization of these MAMs, these proteins can be classified into the following three groups: (1) MAM-localized proteins that are only located at the MAM, (2) MAM-enriched proteins that can also be detected in other regions of the cell, and (3) MAM-associated proteins that are transiently found in MAM in a condition-dependent manner [29]. Because of the high dynamics of MAM, the detailed feature of its components has remained elusive.

The IP3R1-GRP75-VDAC1 complex was the first identified tethering complex. It consists of ER-residing IP3R1s and VDAC1 at the OMM [30]. The OMM-localized FUNDC1 directly binds to IP3R2 to form a bridge between the ER and the mitochondria, favoring Ca^2+^ flux to the mitochondria by enhancing the mitochondria-ER connection in cardiomyocytes [31]. The loss of FUNDC1 promotes IP3R2 ubiquitination and degradation and decreases the levels of the MAM-maintenance protein PACS2 [32]. Additionally, the interaction between IP3Rs and VDAC1 is monitored by GRP75, which maintains the conformational stability of IP3Rs that participate in Ca^2+^ transport from the ER to the mitochondria [30]. The increased IP3R expression level has been found in cardiac hypertrophy, failing myocardium, atrial fibrillation, ischemic dilated cardiomyopathy, and hypertension (Table 1) [33]. Through the IP3R-VDAC complex, Sig-1R participates in cardiac function regulation by interacting with other endoplasmic reticulum chaperones, as the Binding Ig Protein (BiP) forming Ca^2+^-sensitive complexes that extend ionic signaling from ER to mitochondria [34]. The interaction of Sig-1R with lipids is important for their localization and enrichment in MAMs. In skeletal muscle, PDK4 induces the formation of MAMs by interacting with the GRP75-IP3R-VDAC complex at MAMs [35].

**Figure 2:**
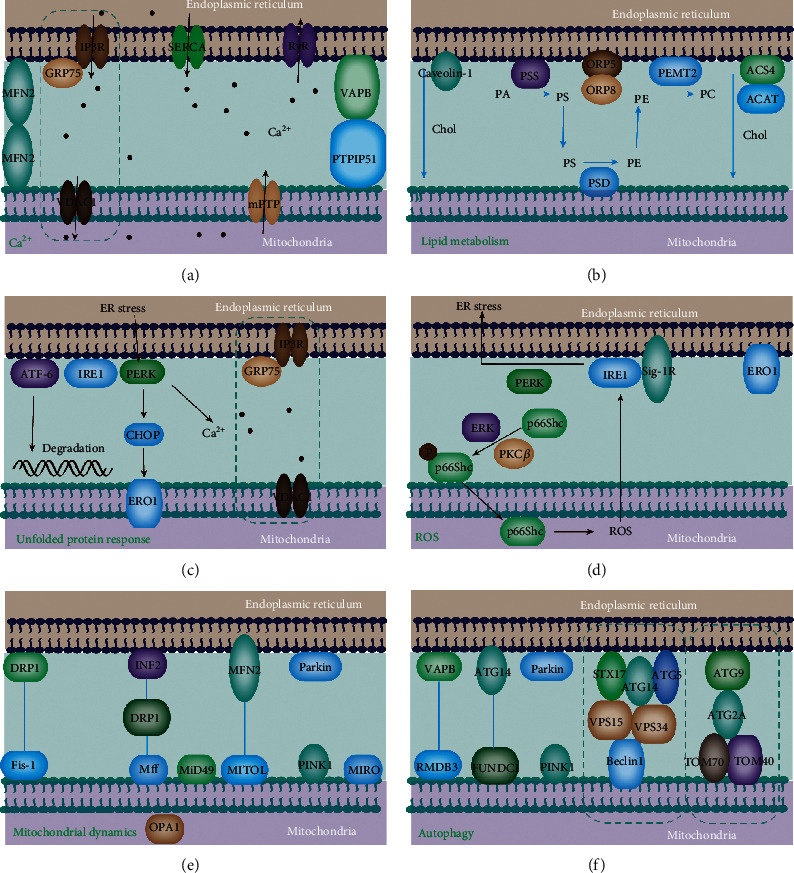
Key cellular functions handled at mitochondria-ER contact sites. Key proteins are involved in related cellular processes and their regulatory mechanisms. (a) Calcium homeostasis regulation. (b) Lipid metabolism. (c) Unfolded protein response. (d) ROS regulation. (e) Mitochondrial dynamics. (f) Autophagy.

MFN2 is known for its role in mitochondrial fusion. It is localized at ER and mitochondrial membranes and forms a hetero- or homodimer with mitofusin-1 (MFN1) or another MFN2 in the outer mitochondrial membrane (OMM) [36, 37]. MFN2 knockdown increases MAM and Ca^2+^ transfer, implying the notion that MFN2 is not a physical tether [38]. Recently, researchers found that loss of MFN2 causes a decrease in the distance between ER and mitochondria membranes, impairing Ca^2+^ uptake into the mitochondria (Figure 2(a)) [39]. Overall, MFN2 is recognized as a key component of MAMs and is crucial for the proper functioning of MAMs. Downregulation of MFN2 has been observed in rat models of cardiac hypertrophy, including spontaneously hypertensive rats, transverse aortic banding, and myocardial infarction, which contribute to cardiomyocyte remodeling. Contrary, its upregulation ameliorates the cardiac hypertrophy induced by angiotensin II [40]. Furthermore, MFN2 is crucial for cardiac differentiation in embryonic stem cells [41].

The BAP31-Fis1 complex consists of ER-localized BAP31 and OMM-localized Fis1 [42]. The BAP31-Fis1 complex is responsible for the recruitment and activation of procaspase 8 and transmission of proapoptotic signals from the mitochondria to the ER. BAP31 is cleaved to form proapoptotic p20BAP31, which transmits calcium from ER to mitochondria and the apoptotic signal via the IP3 receptor complex at ER-mitochondria juxtapositions [43]. Besides, through interaction with TOM40, mitochondrial respiratory chain complexes, and NADH ubiquinone oxidoreductase (mitochondrial complex 1) core subunit 4 (NDUFS4) located on MAMs, BAP31 modulates mitochondrial oxygen consumption and autophagy and maintains mitochondrial homoeostasis [43]. These data imply that BAP31 is a required platform for the transmittance of apoptotic signals between ER and mitochondria. BAP31 is also involved in the attenuated sepsis-mediated myocardial depression by melatonin [42]. Recent data have suggested that synaptojanin-2-binding protein (SYNJ2BP) localized on the OMM interacts with ribosome-binding protein 1 (RRBP1), and once SYNJ2BP is overexpressed, the mitochondria-ER contacts dramatically increase [44].

The VAPB-PTPIP51 complex is composed of ER-residing protein VAPB, which is responsible for vesicle trafficking and the unfolded protein response, and PTPIP51, a protein in OMM modulating cellular development and tumorigenesis [45]. The aberrant interactions between VAPB and PTPIP51 may directly result in a decrease in MAMs and disturbance of Ca^2+^ handling, further leading to a delay in mitochondria Ca^2+^ uptake [46]. PTPIP51 or VAPB alterations are also concomitant with the coverage changes of MAMs on mitochondrial surface. Furthermore, the mutant VAPB also leads to the accumulation of the mitochondria and decreases Ca^2+^ handling [46]. Other proteins can modulate the interaction between PTPIP51 and VAPB complex. A mutation in *α*-synuclein disrupts the VAPB-PTPIP51 complex, contributing to the uncoupling of ER-mitochondria contacts, aberrant Ca^2+^ transfer, and reduced mitochondrial ATP production in the process of Parkinson's disease [45]. TAR DNA-binding domain protein 43 (TDP-43), as a highly conserved and widely expressed nuclear protein, is also responsible for the regulation of the VAPB-PTPIP51 complex [47]. TDP-43 accumulation reduces binding in the VAPB-PTPIP51 complex and disrupts Ca^2+^ homeostasis by promoting the phosphorylation and activation of glycogen synthase kinase-3*β* (GSK3*β*) [47, 48]. Besides, OMM-residing PTPIP51 connection with oxysterol-binding protein-related protein 5/8 (ORP5/8) at the ER membrane facing the cytosol promotes the transportation of the phosphatidylserine (PS) to the mitochondria. Thus, we suppose that MAMs greatly influence the normal physiological function of mitochondria, which can be modulated by the VAPB-PTPIP51 tethering complex [46]. More recently, the ER-anchored MOSPD2 has been proposed as a tethering protein that interacts with PTPIP51 and functions in both intracellular exchange and communication [49].

The GRP78-WASF-ATAD3A complex consists of cytoplasm-localized protein WASF3, the inner mitochondrial membrane (IMM) protein ATPase family AAA domain-containing 3A (ATAD3A), and the ER protein GRP78, by penetrating the OMM and binding to ATAD3A at its N-terminal region [50]. This newly constituted mitochondria-ER tethering complex induces cell invasion in breast and colon cancer [50, 51]. ATAD3A also interacts with OMM and ER-resident proteins, including MFN2, dynamin-related protein 1 (Drp1), and BiP via the cytosolic protein WASF3 [52]. Instead of the above-discussed protein tethers, MAMs harbor a wide variety of regulatory proteins. TG2 modulates Ca^2+^ flux and protein composition through interaction with GRP75 [53].

For obtaining a more comprehensive knowledge of proteins in maintaining ER-mitochondria contacts, identification of additional tethers or spacers is essential. Further studies related to single-protein ablation should evaluate whether the activity or the localization of MAMs is the proper method for identification of cooperating protein complexes.

## 3. The MAM-Mediated Regulation of Cellular Homeostasis in the Cardiovascular System

**Figure 3:**
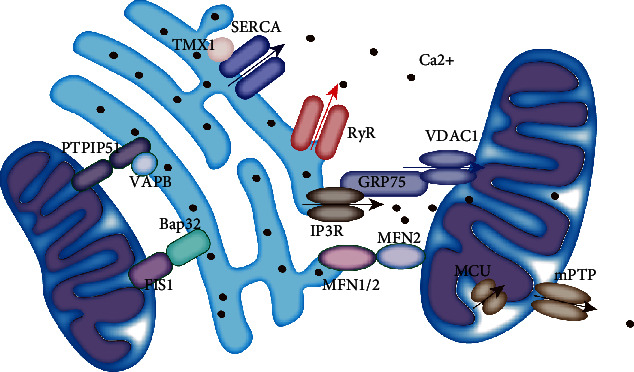
Components of MAMs in the cardiovascular system. Graphic representation of the proteins that are part of the MAMs in the cardiovascular system.

MAMs participate in various cellular processes, such as calcium homeostasis, lipid metabolism, mitochondrial physiology, mitophagy, ER stress, and inflammation [54]. The proteins enriched in the cardiomyocyte MAMs and their role in MAM-regulated processes are shown in Figure 3. The BAP31-Fis1 complex, PTPIP51-VAPB complex, MFN1/2 complex, IP3R-GRP75-VDAC1 complex, and SERCA-TMX1 were involved in the cardiovascular system. The detailed functions of these complexes were discussed in the following sections.

## 4. Calcium Transfer

Ca^2+^ transfer between organelles seems to affect both the heart and the vascular system [25, 55]. During ischemia and reperfusion, mitochondria calcium increases accompanied with mitochondrial permeability transition pore (mPTP) activation. Sarcoplasmic reticulum (SR), formed by ER, is a membrane-bound structure existing in muscle cells (myocardium and skeletal muscle), similar to ER in other cells [28]. The main function of SR is to store Ca^2+^. SR releases Ca^2+^ in response to electrical stimulation or pharmacological activation of RyR and increases mitochondrial Ca^2+^ level [56]. Ca^2+^ is free to enter the outer mitochondrial membrane with the aid of VDAC1. However, the inner mitochondrial membrane is not permeable, and calcium can enter the inner mitochondrial membrane only through a mitochondrial calcium uniporter (MCU) channel [30]. Therefore, MCU may have a marked impact on cardiac myocyte metabolism and function.

Ca^2+^ transmission from the ER to mitochondria is involved in mitochondrial apoptosis and energy generation with the aid of MAMs (Figure 2(a)). IP3R3-GRP75-VDAC1 complex is the main channel responsible for Ca^2+^ release from the ER to mitochondria [30]. IP3R1 forms a high concentration of Ca^2+^ in the vicinity of ER. The VDAC1 functions as a Ca^2+^ uptake channel in the OMM. GRP75 connects two channels through their cytosolic domains to form VDAC1/GRP75/IP3R1 channel complex [25]. The IP3R3-GRP75-VDAC1 complex also acts as a molecular scaffold for other calcium-handling players (Sig-1R, BiP, Bcl-2, and IRBIT), which are essential for the precise regulation of the calcium signaling through the IP3R3-GRP75-VDAC1 axis at the MAM (Figure 2(a)) [57, 58]. The increased level of IP3R expression has been observed in cardiac hypertrophy, failing myocardium, atrial fibrillation, ischemic dilated cardiomyopathy, and hypertension, suggesting its contribution to the development of cardiac hypertrophy [31, 32]. Additionally, IP3Rs could modulate excitation-contraction coupling both in ventricular and atrial cardiomyocytes [33]. Sig-1R maintains the stability of IP3R to ensure appropriate Ca^2+^ signaling between the ER and mitochondria [34]. Interaction of VAPB with PTPIP51 facilitates ER-mitochondria Ca^2+^ exchange (Figure 2(a)).

RNA-dependent protein kinase- (PKR-) like ER kinase (PERK) as a key ER stress protein is involved in calcium regulation, the maintenance of ER morphology, and MAM construction [59]. In addition, calnexin (CNX), which is involved in protein folding, interacts with ER calcium pumps. Deficiencies in these proteins cause obvious reduced mitochondrial Ca^2+^ import and impairment in Ca^2+^ dynamics [60]. The homeostasis of mitochondria Ca^2+^ is closely related with mitochondrial ATP production [61]. Excess transfer of calcium from ER to mitochondria induces mitochondrial Ca^2+^ overload and oxidative stress [62]. On the contrary, when the ER-mitochondria interaction is weakened, excess calcium will release to the cytosol, leading to cytosolic Ca^2+^ wave. Recently, transient receptor potential cation channel (TRPM8) was identified as a compartment in the maintenance of cellular and mitochondrial Ca^2+^ in the vascular smooth muscle (VSMC) cells [63]. Activation of TRPM8 relieved mitochondrial respiratory dysfunction and excess ROS generation induced by angiotensin II by preserving mitochondrial Ca^2+^-dependent PDH activity, thus lowering blood pressure in cold or in angiotensin II-induced hypertensive mice [63]. However, the exploration of other TRP channels in MAM responsible for calcium transportation from ER to mitochondria remains to be further illustrated.

## 5. Lipid Synthesis and Exchange

Lipid molecules are involved in multiple cellular processes, such as cell membrane formation, cell signaling transduction, and synaptic transmission [64, 65]. While ER has a paramount role in lipid synthesis, other organelles' assistance is essential since several of the key enzymes are located on the membrane of organelles, such as mitochondria [66]. MAMs tethering proteins involved in phospholipid synthesis and transport include diacylglycerol O-acyltransferase 2 (DGAT2), fatty acid CoA ligase 4 (FACL4), phosphatidylethanolamine N-methyltransferase 2 (PEMT2), cholesterol acyltransferase/sterol O-acyltransferase 1 (ACAT1/SOAT1), and PSS1 and PSS2 with an ascribed function as a platform for lipid biosynthesis and exchange (Figure 2(b)) [65]. FACL4 is considered one of the most reliable MAM markers and is responsible for synthesizing triacylglycerol [67]. ACAT1, as another mitochondrial enzyme related to lipid metabolism, catalyzes the cholesteryl ester formation from free cholesterol, maintaining the dynamic balance between membrane-bound and cytoplasmic lipid droplet stored cholesterol [68]. MAMs are identified as cholesterol-rich membranes characterized by the sterol-interacting protein caveolin. MAM-associated caveolin-1 (CAV1) is involved in cholesterol efflux through its interaction with VDAC2, and silencing CAV1 in liver MAM leads to aberrant intracellular free cholesterol accumulation, as well as the reduced physical extension and integrity of MAM (Figure 2(b)) [69]. Ablation of the CAV1 gene aggravates cardiac dysfunction and decreases survival in mice exposed to myocardial ischemia [70]. Moreover, inhibition of *CAV1* and *ApoE* in mice increased protection against atherosclerotic lesions compared to *ApoE*^−/−^ mice [71].

Additionally, MAM-enriched proteins induce the formation of the main structural component of biological membranes, phosphatidylcholine, PE, and phosphatidylserine. Phosphatidylserine is first synthesized by PSS1 and PSS2 in the MAM, after which it is transferred to the tightly connected mitochondria via the MAMs' lipid transfer tethering ORP5/8-PTPIP51 [72]. It is converted into phosphatidylethanolamine (PE) in the inner mitochondrial membrane by a decarboxylase. The newly generated PE is then transferred from the mitochondria, where it is methylated by the MAM-enriched PEMT2 to generate phosphatidylcholine (PC), a major component of the cell membrane. PC must be transferred back to mitochondria once again since it is a component of the mitochondrial membrane. The phospholipid acids are synthesized in ER and transferred to mitochondria to modify cardioprotective mitochondrial cardiolipin, which is necessary for stability and activity of mitochondrial Ca^2+^ uniporter (Figure 2(b)) [73]. The level of cholesterol esters, PEs, and triacylglycerols are closely related to cardiovascular diseases. MAMs are also involved in the production of ceramide, a bioactive sphingolipid that is important for regulating cell growth arrest, differentiation, apoptosis, and inflammation.

The changes in MAM-maintaining proteins affect lipid anabolism. The HDL cholesterol and phospholipids have shown to be significantly elevated in ORP8-depleted mice [74]. Additionally, primary hepatocytes with ORP8 deficiency produce nascent HDL particles, suggesting changed HDL biosynthesis [74]. *ATAD3* gene cluster deletions in human fibroblasts perturbed cholesterol and lipid metabolism [53]. Human MFN2 exerts antiatherogenic properties in a rabbit model of atherosclerosis, while MFN2 overproduction leads to reduced VSMC proliferation/hyperplasia and diminished plaque progression [75]. PACS2 depletion diminishes the levels of the fatty acid metabolism enzymes PSS1 and FACL4 in human skin melanoma cells [76]. Thus, PACS2 might be a promising therapeutic target for atherosclerosis by having an important role in oxidized low-density lipoprotein- (OxLDL-) induced EC apoptosis, perturbing MAM formation and mitochondrial Ca^2+^ elevation.

## 6. MAM Modulates ER Stress

ER stress occurs when the misfolded proteins aggregate in the lumen and the homeostasis of ER is disrupted [77]. ER stress is mediated by the ER-localized sensor protein kinase PERK, ATF6, and IRE1*α*, which are maintained in an inactive form by GRP78 [78]. GRP78 is upregulated in the heart under multiple cardiac pathological conditions, such as dilated or ischemic cardiomyopathy. The activation of these proteins triggers an ER-specific UPRl, which is strongly induced by myocardial ischemia. ER stress signaling is modulated by MAM-tethering proteins since the activity of PERK is repressed by MFN2 by binding (Figure 2(c)) [79]. PERK is involved in the maintenance of the mitochondria-ER contacts and enhancement of the ROS-induced mitochondrial apoptosis. Once MFN2 is depleted, PERK is activated, and the PERK-EiF2*α*-ATF4-CHOP pathway is enhanced (Figure 2(c)) [80]. Studies have suggested that ATF6 interacts with the tethering protein VAPB to suppress the UPR directly. ATF6 transcription is attenuated by VAPB overexpression in HEK293 and NSC34 cells [78]. IRE1*α* accumulation in MAMs either leads to cell survival by splicing the *Xbp1* mRNA or cell death induction by promoting mitochondrial Ca^2+^ overload (Figure 2(c)) [78]. Ubiquitylation of IRE1*α* at MAM could hinder ER stress-induced apoptosis [81]. Cardiac-specific *Xbp1* knockout mice (cKO) exhibited a significant increase in myocyte death and more profound pathological remodeling in cKO mice, thus suggesting that induction of Xbp1 is necessary to protect the heart from ischemia/reperfusion (I/R) injury *in vivo*. In addition, depletion of other MAMs, such as PACS2, Sig-1R, MFN2, or CypD triggers ER stress by disrupting the ER-mitochondria communication. In contrast, mitochondria-ER contacts could also be modulated by the ER stress proteins. The ER-mitochondria contacts mediated by an initial ER stress enhance mitochondrial ATP production and Ca^2+^ uptake, thus leading to cellular adaptation to ER stress.

### 6.1. Regulation of Oxidative Stress

Under normal conditions, ROS is produced at physical levels, which is necessary for the maintenance of cellular homeostasis [82]. The production of excessive ROS, especially mitochondrial ROS (mtROS), can lead to oxidative damage to proteins, lipids, and DNA, which eventually leads to CVD [83]. MAM-mediated excessive Ca^2+^ transfer is reported to promote mtROS generation. For instance, diabetes promotes the formation of MAMs in podocytes, resulting in elevation of Ca^2+^ transfer from the ER to mitochondria, finally leading to mtROS overgeneration. However, MAM formation suppression induced by FUNDC1 depletion alleviated mtROS production. These studies confirmed the relationship between MAM alterations and mtROS excess (Figure 2(d)) [31]. Also, several Ca^2+^ channel regulators in MAMs were found to regulate Ca^2+^ and MAM-dependent mtROS generation. For instance, MAMs highly enriched ER representative oxidoreductases, oxidoreductin-1*α* (Ero1*α*), and endoplasmic reticulum resident protein 44 (ERp44) can trigger the excess production of mtROS [84]. Mechanically, Ero1*α* induces IP3R1 oxidation, leading to the dissociation of ERp44 from IP3R1, thereby enhancing the transfer of Ca^2+^ from the ER to mitochondria, contributing to excess mtROS production [85]. Selenoprotein N (SEPN1), a type II transmembrane protein of the ER, is enriched in MAMs and senses luminal calcium with its EF-hand domain [86]. When ER calcium is consumed, SEPN1 regulates ER calcium supplements mediated by the SERCA, protecting cells from ER stress. SEPN1 also resists oxidation elicited by Ero1*α*, leading to the inactivation of SERCA. SEPN1 depletion triggers decreased ER-mitochondria contacts, reduced organelle Ca^2+^ content, and damaged OXPHOS [86].

Glutathione peroxidase 8 (GPX8), another type II transmembrane protein, also localized at MAMs, has a role in the modulation of Ca^2+^ dynamics [87]. In particular, GPX8 overexpression causes a decreased ER calcium coupled with a reduction of the ER-mitochondria calcium flux [88]. ER chaperones and folding assistants modulate cell metabolism and survival by controlling ER-mitochondria calcium flux. MAMs could also regulate the production of mtROS via DsbA-L and p66Shc [89]. DsbA-L, a multifunctional protein, is localized in the mitochondrial matrix, ER, and the MAM fraction, which is also closely related to the production of mtROS. The positive regulation of p66Shc in mtROS production and mitochondrial fission was well established and largely dependent on Ser36 phosphorylation. Notably, p66Shc phosphorylation also initially translocates p66Shc to the MAM fraction, therefore participating in mtROS production [90]. Further analysis revealed that the transfer of p66Shc to the MAMs correlates well with the production of mtROS (Figure 2(d)).

In summary, these findings imply that MAMs have a key role in the maintenance of the mitochondrial redox state and, consequently, in the homeostasis of cellular redox. However, the detailed function of MAMs in ROS excess production and its function in the occurrence and progression of CVD are not fully understood and need to be further investigated.

## 7. MAM Regulates Mitochondrial Physiology

In addition to the modulation of mitochondrial Ca^2+^ transport, MAM also affects mitochondrial physiology, including mitochondrial bioenergetics, dynamics, and mitophagy (Figure 2(e)). Since the heart is an energy-hungry organ and heart muscle cells are rich in mitochondria, the intact mitochondrial homeostasis is essential for the heart functions such as contractile function and cardiomyocyte metabolism, and the dysfunction of mitochondrial dynamics ultimately results in various CVDs. Mitochondrial dynamics include mitochondrial fission, fusion, and motility.

### 7.1. Mitochondrial Fission

During fission, Drp1 forms a helix around mitochondria that constricts and divides the mitochondrion into two parts after it is recruited to the OMM. The recruitment of Drp1 is dependent on its receptors, such as Fis1, mitochondrial fission factor (Mff), and mitochondrial dynamics proteins of 49 and 51 kDa (MiD49/MiD51), which are localized at the ER-mitochondria interface before mitochondrial fission (Figure 2(e)) [91]. The binding of Drp1 to F-actin *in vitro* stimulates the oligomerization and GTPase activity of Drp1, subsequently enabling Drp1 to spiral around the preconstricted mitochondria, thus mediating their fission [92]. As a new mitochondrial receptor for Drp1, FUNDC1 promotes mitochondrial fission by interacting with the ER-resident protein calnexin in response to hypoxia [32]. However, even in the absence of Drp1, mitochondrial constriction can also form in the vicinity of mitochondria-ER contacts, implying that ER tubules may precede mitochondrial fission and define the position of mitochondrial division sites. Mice with a Drp1 mutation develop cardiomyopathy along with spotty calcifications in heart tissue [93]. Other proteins involved in mitochondrial fission regulation, such as inverted formin 2 (INF2), syntaxin 17 (STX17), and Rab32, were also detected at the MAM. For instance, INF2 launches mitochondrial constriction and division through binding, actin-nucleating protein Spire, Spire1C (Figure 2(e)) [94]. STX17 l induces mitochondrial fission by determining Drp1 localization and activity. [93]. Mechanistically, decreasing MAM formation reduces the Ca^2+^ concentrations in both mitochondria and cytosol. Decreasing intracellular Ca^2+^ levels can repress the binding activity of cAMP response element-binding protein (CREB) to the *Fis1* promoter, thus inhibiting Fis1 expression and mitochondrial fission. Upregulation of dynamin-related protein 1 (Dnp1), a fission protein, leads to fragmentation of mitochondria and heart ischemia. The formation of proapoptotic complex with MFN2 and Bax on the OMM is accompanied by the mPTP opening, cytochrome c (Cyto C) release, mitochondrial fragmentation, and cardiomyocyte apoptosis induction [95]. Inhibition of Dnp1 may protect cardiac mitochondria from fragmentation and prevent apoptosis.

### 7.2. Mitochondrial Fusion

Mitochondrial fusion is mainly modulated by outer mitochondrial membrane protein MFN and the IMM protein optic atrophy 1 (OPA1). MFN2 promotes mitochondrial tethering and mitochondrial fusion [96]. *MFN2* null hearts in adult mice showed better recovery from ischemia/reperfusion injury compared to *MFN2*-floxed controls [97]. Besides, MFN2 tethering with the mitochondrial ubiquitin ligase MITOL regulates mitochondrial dynamics (Figure 2(e)) [98]. Mitochondrial fusion facilitates the content exchange between mitochondria and restores impaired mitochondria. A recent study proposed that the fission and fusion processes initiate at the same ER-mitochondria contact site for the maintenance of mitochondrial morphology in response to external insults and metabolic cues, such as the nutrient availability, while MFNs also accumulate at the MAMs where fusion occurs [99]. However, it remains unclear how the positions of mitochondrial fusion sites are determined. Both membrane fission and fusion are considered to be promoted by the specialized lipid environment of the MAM [100]. In addition, the high Ca^2+^ condition favors the initiation of fission and fusion. Moreover, it is still unclear whether interrupted ER-mitochondria contacts cause mitochondrial elongation or mitochondrial fragmentation. A previous study mentioned reduced mitochondrial and cytosolic Ca^2+^ levels in response to decreased MAM formation; decreased intracellular Ca^2+^ concentration repressed Fis1 expression and mitochondrial fission, leading to mitochondrial elongation [101]. In contrast, Tian et al. observed increased cytosolic Ca^2+^ levels in response to defective ER-mitochondria contact, indirectly activating Drp1 by means of activating calcineurin phosphatase and subsequently leading to mitochondrial fragmentation [93]. Although the mechanism for this contradiction remains unclear, we suppose the integrity degree of MAMs may be a relevant factor. Consequently, further investigations should focus on the distance between the ER and mitochondria for a better understanding of the relationship between alterations in mitochondrial morphology and ER-mitochondria contact.

### 7.3. Mitochondrial Motility

The mitochondria are transferred to satisfy local energy demands and Ca^2+^ buffering demands. The transportation along microtubules is associated with mitochondrial Rho GTPase 1 (MIRO1) and MIRO2 [102]. The relation of MIRO1/2 in the involvement of mitochondria motility is supported by previous studies [103]. However, due to the low calcium affinity of MIRO1/2, the binding requires a high Ca^2+^ concentration, making MAMs an ideal site for mitochondria redistribution [104]. Also, excess ER-mitochondria contact and Ca^2+^ transfer may result in defects in axonal mitochondrial transport. Microproteins, Ca^2+^-sensitive GTPases localized on the OMM, interact with the TRAK adaptors and dynein/kinesin motors for the regulation of microtubule-dependent transport of the mitochondria [105]. The motor protein KIF5B was found to actively deliver mitochondrial DNA (mtDNA) nucleoids promoted by ER-mitochondria contacts via mitochondrial dynamic tubulation [106]. Mic60 on the mitochondrial inner membrane seems to link mitochondrial nucleoids to the mitochondrial outer membrane protein Miro1 and KIF5B at MAMs [107]. Such active transportation is an essential mechanism for the proper distribution of nucleoids in the cell's peripheral zone.

## 8. MAMs and Autophagy

MAMs are a crucial compartment for the induction and execution of autophagy. Many autophagy-related gene (ATG) proteins are enriched at MAMs, including ATG14 (autophagosome marker), ATG2/5 (autophagosome-formation marker), double FYVE domain-containing protein 1 (DFCP1, a platform for autophagosome formation), Beclin1, and VPS15/34. Moreover, TOM40/70 directs ATG2A to MAMs to mediate phagophore expansion (Figure 2(f)) [108]. On the MAMs, ATG2A directs the delivery of ATG9-vesicle to stimulate phagophore expansion and efficient autophagic flux (Figure 2(f)) [109]. Impaired ER-mitochondria connection induced by PACS2 and MFN2 knockdown efficiently inhibits the formation of ATG14 puncta. Also, the formation of autophagosomes could be modulated by promyelocytic leukemia protein (PML), a tumor suppressor, localized at the MAMs by regulating the activity of the AMPK/mTOR/ULK1 pathway via affecting the transport of calcium ions from the ER to mitochondria [110]. Mitophagy removes impaired and dysfunctional mitochondria. Mitophagy occurs at the MAM site among species [111]. The mitophagy process is composed of the following six steps: autophagy induction, the isolation membrane nucleation (also known as the phagophore), the isolation membrane expansion, the autophagosome formation, the autophagosome fusion with a lysosome to form an autolysosome. Two major pathways were reported to be involved in mitophagy induction: (1) Parkin-dependent pathways, composed of Parkin and PTEN-induced putative kinase1 (PINK1) (Figure 2(f)), and (2) adaptor-dependent mitophagy, mediated directly by Bcl2 interacting protein 3 (BNIP3), Bcl2 interacting protein 3 like (BNIP3L/NIX), and FUNDC1, which possess an LC3 interacting region (LIR) and interact with light chain 3 (LC3) to mediate mitophagy (Figure 2(f)) [112–114]. Both mitophagy pathways were accordant with MAMs. Mitophagy protects myocardial cells from I/R injury. I/R reduces mitophagy and stimulates apoptosis in cardiomyocytes. An appropriate increase in mitophagy potentially alleviates I/R-induced cardiomyocyte apoptosis. Simultaneously, mitophagy may also have a negative role in I/R injury [114]. Inhibition of mitophagy potentially protects the myocardium against I/R injury, reduces cardiomyocyte apoptosis, improves cardiac function, and protects mitochondrial integrity.

### 8.1. PINK/Parkin-Mediated Mitophagy

The PINK1 and Parkin pathway, one of the best known and most well-studied pathways of mitophagy, is implicated in the progression of Parkinson's disease [113]. Under physiological conditions, PINK is continuously delivered to the mitochondria and degraded by matrix processing peptidases (MPPs). The degraded product is then incised by presenilin-associated rhomboid-like (PARL). Then, cleaved PINK is transferred to the cytoplasm for eventual degradation in lysosomes [115]. Under pathological conditions, the cleavage of PINK is blocked due to defective mitochondrial function. The noncleaved PINK accumulates on the mitochondria's outer membrane through a process of translocation of the OMM protein to the outer membrane (TOM). In addition, the aggregation of PINK on the OMM can also be affected by the level of mitochondrial pyruvate by facilitating the direct interaction between PINK1 and TOM [114]. The accumulation of PINK on the OMM leads to the phosphorylation of ubiquitin, thereby recruiting Parkin. Subsequently, PINK phosphorylates and activates Parkin on the OMM and then activates Parkin polyubiquitinate proteins, such as VDAC1 and p62/SQSTM1. The binding of ubiquitinated substrates and autophagic membrane proteins, LC3 via LIR motif, promotes recruitment of the autophagosomal membrane around the mitochondria. The normal signaling pathway of PINK/Parkin-mediated mitophagy is a fundamental process for the homeostasis of intracellular mitochondria, and defects in this process may lead to many diseases, such as CVD [116]. A previous study reported that PINK1 and Parkin were upregulated and enhanced Parkin transfer and activation during I/R injury accompanied with BNIP3 upregulation and FUNDC1 downregulation [112]. *PINK1* knockout mice were more susceptible to overload-induced heart stress and consequent heart failure compared to wildtype mice.

BECN1, a key component of the class III phosphatidylinositol 3-kinase (PtdIns3K) complex, is present in MAMs, where it enhances the ER and mitochondria contacts and initiates the formation of autophagosome precursors [113]. Thus, PINK/Parkin-dependent mitophagy initiates at the site of MAMs. Loss of PINK inhibits the accumulation of BECN1 at MAMs, which is PARK independent, thus suggesting a novel function of PINK in regulating mitophagy. BECN1 is aberrantly expressed or posttranslationally modified in many heart diseases, including ischemia/reperfusion, myocardial infarction, cardiac hypertrophy, and heart failure [117]. BECN1 deregulation leads to heart disease development through altered myocardial autophagy and apoptosis [110]. Furthermore, Glycoprotein 78 (Gp78), a MAM-located ubiquitin ligase (E3), has been reported to be involved in mitophagy. The existing evidence implies that the core proteins related to PINK/Parkin-mediated mitophagy are enriched in MAMs and are responsible for the regulation of MAMs' integrity and function. As a driver of Parkin-mediated mitophagy, AMPK has a protective role in cardiac functions [110]. The hypoxic injury and myocardial infarction (MI) are associated with increased RIPK3 expression, leading to inactivation of AMPK.

### 8.2. FUNDC1-Mediated Mitophagy

In mammalian cells, FUNDC1, a highly conserved protein, is involved in the receptor-mediated mitophagy pathway [118]. During hypoxia, the LC3-binding regions (LIR) domain of FUNDC1 located in the mitochondrial outer membrane recruits LC3 to induce mitophagy. FUNDC1-related mitophagy is modulated by a variety of stress factors and cellular proteins [119]. Under normoxic conditions, FUNDC1 can be phosphorylated by Src and casein kinase 2 (CK2), respectively, inhibiting the binding with LC3 to initiate autophagy [120]. During hypoxic conditions, FUNDC1 is dephosphorylated by the mitochondrial protein phosphatase PGAM5, resulting in the binding of FUNDC1 to LC3 to induce the formation of autophagosomes [121]. ULK1, a Ser/Thr kinase, mediates the formation of early autophagosomes and is closely associated with FUNDC1-dependent mitophagy [122]. ULK1 expression level is increased and recruited to the fragmented mitochondria under hypoxia or treatment with FCCP. In addition, transferred ULK1 interacts with FUNDC1 to promote the phosphorylation FUNDC1, thereby initiating autophagy [123]. MARCH5, a mitochondrial E3 ligase, is involved in the regulation of mitophagy; MARCH5 can directly interact and degrade FUNDC1 by inducing its ubiquitination [124].

FUNDC1-mediated mitophagy is confirmed as directly related to MAMs via binding to IP3R2 to mediate IP3R-dependent Ca^2+^ signaling from ER to mitochondria and cytoplasm [125]. When FUNDC1 expression level is decreased, the intracellular Ca^2+^ decreases, and Fis1 expression level is inhibited through Ca^2+^-sensitive CREB, leading to mitochondrial dysfunction [126]. In addition, the reduced expression level of FUNDC1 impaired the interaction between the ER and mitochondria and reduced the protein abundance in MAMs. Under normoxic conditions, the accumulation of FUNDC1 in MAMs remains low in content, while under hypoxia conditions, FUNDC1 substantially aggregates in MAMs.

CNX may be indispensable during this process. The N terminus of CNX directly binds to the hydrophilic domain of FUNDC1. However, due to the localization of the N terminus of CNX in the lumen of the ER, there must exist an unknown protein that mediates the interaction between CNX and FUNDC1 [127]. Under hypoxic conditions, CNX depletion represses FUNDC1 translocation to the MAMs, further confirming the vital importance of CNX in the translocation of FUNDC1. Despite the requirement of further study on the function of MAMs in FUNDC1-mediated mitophagy, existing evidence suggests that MAM offers a platform for FUNDC1 to exert its biological functions. FUNDC1 is required for cardiac ischemia/reperfusion injury-activated mitophagy. Hypoxic pretreatment induces FUNDC1-dependent mitophagy in platelets and reduces I/R-induced heart injury [128]. Another research also mentioned that MAM-localized STX17 could bind to ATG14, an autophagosome marker, and transfer it to the MAM until the completion of autophagosome formation. This supports the idea that the autophagosome forms at MAMs.

## 9. Other Functions of MAMs

### 9.1. Apoptosis

Apoptosis is a tightly regulated, cellular deletion process found in various cardiovascular diseases, such as myocardial infarction, reperfusion injury, and heart failure [42]. Excess transfer of calcium leading to calcium overload can induce cell apoptosis. Specifically, mitochondrial calcium overload induces mPTP opening, IMM permeabilization, mitochondrial depolarization, and finally cell apoptosis. The binding between Fis1 to BAP31 contributes to ER-mitochondria tethering in the process of apoptosis [129]. Also, the formation of the Fis1–BAP31-tethering complex can be induced by exogenous inducers of apoptosis [43]. Consistent with the previous data, cell apoptosis-related to Fis1 overexpression is associated with the excessive ER-mitochondria Ca^2+^ transfer [130].

The relationship between cell apoptosis and mitochondrial dynamics is not fully understood. After the induction of the cell apoptosis process, massive Drp1 is recruited to the OMM, leading to an increase of mitochondrial network fragmentation [93]. In line with this data, a dominant-negative mutation in Drp1 inhibits cell apoptosis. Contrary, Drp1 overexpression protects against Ca^2+^-mediated apoptosis. Therefore, the function of Drp1 in Ca^2+^-induced apoptosis is still controversial. In addition, Drp1 induces cell apoptosis by facilitating oligomerization of Bax under apoptotic conditions [101]. The mechanism of Bax in apoptosis is similar to Drp1 [92]. The speculation that MAMs are involved in this programmed cell death is confirmed by the fact that Bax oligomerization on the OMM is conducted by key lipid effectors of Bax between ER and mitochondria [131].

Knowing that Bax and MFN2 are colocalized in foci during apoptosis, it is speculated that MFN2 functions in OMM permeabilization and in response to apoptotic stimuli [52]. A recent study suggested an induction of Drp1 SUMOylation in response to the activation of apoptosis, during which cytochrome c release is a requisite for the process [91]. The MAM contact sites stabilized by SUMOylated Drp1 act as a place for mitochondrial constriction, calcium flux, cristae remodeling, and cytochrome c release. SUMOylated Drp1 can lead to mitochondrial dysfunction contributing to cardiac hypertrophy. Taken together, these data suggested a complicated network for apoptosis, mitochondrial dynamics, and MAMs, which deserves further studies.

### 9.2. Inflammation

Inflammation has been implicated to be involved in one of the MAM-related pathways [132]. As a cytosolic multiprotein complex, the inflammasome is responsible for the activation of inflammatory responses. The NLRP3 complex is currently the only known MAM-related inflammasome complex [133]. A wide variety of stimuli can be used to activate NLRP3. However, the exact activation mechanism remains unclear.

NLRP3 activation is implicated in mitochondrial dysfunction, the release of mtROS and mtDNA into the cytosol. In addition, enhanced NLRP3 activation is accompanied by increased damaged and dysfunctional mitochondria induced by the inhibitors of mitophagy [134]. NLRP3 activation is consistent with the shift of thioredoxin-interacting protein (TXNIP) from cytosolic thioredoxin 1 to the NLRP3 inflammasome on the MAM. Interestingly, MAM-enriched VDAC is indispensable for inflammation since the elimination of VDAC1/2 abrogates the formation of the inflammatory body. Rats with myocardial infarction exhibited a marked increase of VDAC1 in both ventricular and atrial tissues. VDAC1 inhibition can alleviate excessive fibrosis in the atrial myocardium, which may have important therapeutic implications [112].

Besides the functions in NLRP3 activation, mitochondria may also participate in inflammasome assembly, because of the contact of activated inflammasome with both mitochondria and MAMs. Furthermore, the contact between NLRP3 and mitochondria is mediated by at least three mitochondrial factors: mitochondrial antiviral-signaling protein (MAVS), MFN2, and cardiolipin. The physical interaction between MAVS and NLRP3 is required for NLRP3 inflammasome activation during viral infection [135]. Also, MFN2 directly binds to NLRP3 during viral infection. Cardiolipin is a mitochondrial inner membrane lipid, and has been shown to externalize and directly bind NLRP3; disruption of its expression is harmful for NLRP3 activation [136]. Overall, MAMs are closely associated with inflammation, and further studies are required to understand the mechanisms.

### 9.3. Antiviral Response

The relationship between MAMs and immune response has also been identified during RNA virus infection. Viral infection induces host innate immune responses by activating transcription factors NF-*κ*B and IRF3 that modulate type-I interferons' expression level to inhibit viral replication. RIG-I is one of the retinoic acid-inducible gene- (RIG-) like receptors (RLRs) that detect virus intracellular dsRNA. Once the virus dsRNA is recognized, the cytoplasmic RIG-I translocates to MAMs and interacts with MAVS, activating NF-*κ*B and IRF3 pathways [137]. In addition, MAVS from the MAM can be targeted and cleaved by the viral NS3/4A protease to ablate innate immune signaling during HCV infection, implying that the RIG-I pathway function against HCV is likely coordinated by MAM-resident MAVS [138].

The stimulator of interferon genes (STING) was identified as a positive regulator of RIG-I-associated IFN-*β* signaling [137]. The complex of STING and RIG-I recruits MAVS-induced IRF3 phosphorylation and IFN production in response to RNA and DNA viruses. In addition, STING is a target for HCV-NS4B to block RIG-I-mediated activation of IFN-*β* production by blocking its interaction with MAVS. NS4B and STING are localized on ER and MAM [139]. MAM-resident factor Gp78 was also found to be involved in innate antiviral signaling by targeting MAVS during vesicular stomatitis virus (VSV) infection [140]. Still, the association between MAMs and antiviral response should be further studied to advance the understanding of the potential therapeutics in viral infections.

## 10. MAM Is Widely Involved in the Homeostasis Regulation of the Cardiovascular System

**Figure 4:**
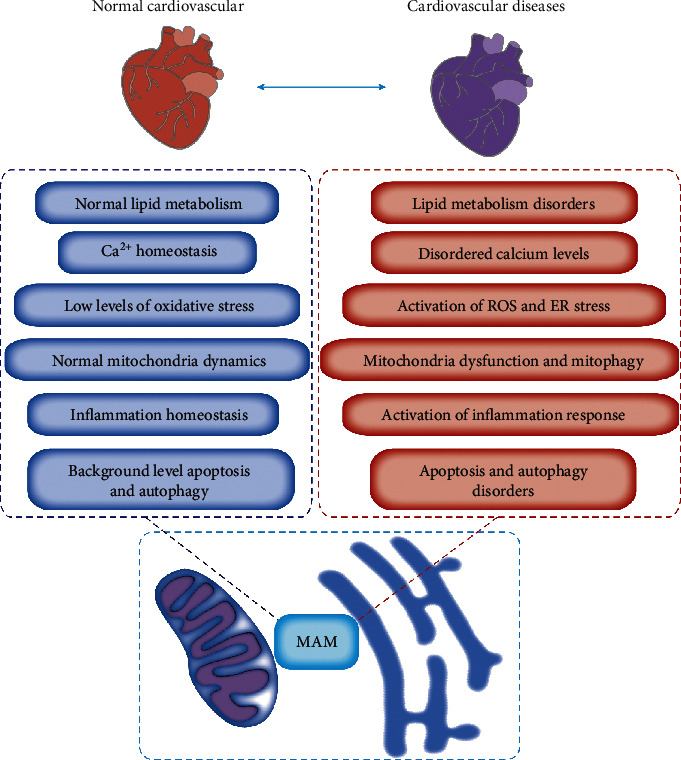
The alteration of MAM-mediated cellular functions in cardiovascular diseases. Major MAM-mediated abnormalities leading to CVD include lipid metabolism disorders, abnormal calcium levels, the activation of ROS and ER stress, the dysfunction of mitochondria and mitophagy, the activation of inflammation response, and the disorders of apoptosis and autophagy.

Once the mitochondrial membrane potential is inhibited or partially inhibited, the calcium kinetics during contraction is altered, accompanied by the enhanced reduction of shortening degree of cardiomyocytes. This result suggested the involvement of calcium transportation from SR to mitochondria in cardiac contraction. The dysregulation between the ER and mitochondria in the pathogenesis of CVD is gradually recognized. The effect of MAMs' dysfunction on CVD is probably due to the involvement of multiple pathways, including lipid metabolism disorders, aberrant calcium levels, activation of ROS and ER stress, mitochondria dysfunction and mitophagy, activation of inflammation response, apoptosis, and autophagy disorders (Figure 4).

Several key MAM-associated proteins affect the progression of CVD. Cardiac-specific *MFN2* knockout mice displayed cardiac hypertrophy and moderate diastolic dysfunction [141]. Under *β*-adrenergic stress conditions, the same mice showed obvious systolic dysfunction. Moreover, MFN2 knockout mice displayed abnormal large and elongated mitochondria morphology and reduced SR and mitochondria contacts. In addition, deficiency in MFN2 in cardiomyocytes resulted in the abnormal spatial distribution of mitochondria, low mitochondrial membrane potential, and reduced Ca^2+^ uptake [142].

The FUNDC1 deficiency reduces the contact in ER and mitochondria [143]. *FUNDC1* knockout mice displayed diastolic and systolic dysfunction. Besides, these mice exhibited obviously lower early and late ventricular filling velocity ratio and decreased ejection fraction [126, 144].

The mitochondrial dynamics in myocardial tissue are an important aspect of cardiac function. *Drp1* knockout mice exhibited a lower beating rate in isolated cardiomyocytes [145]. The potential mechanism may be mitochondrial respiration defects, suppressed autophagy, and increased mitochondrial ROS production [143, 146]. Above all, the normal mitochondrial fission for a myocardial cell can be vital for energy supply. Therefore, cardiac-specific Drp1 ablation mice led to decreased life span, diminished survival, cardiac hypertrophy, fibrosis, and reduced systolic function [93].

Mitochondrial damage, mPTP, and ER stress are considered as the main factors related to the reperfusion damage in I/R. Since MAMs modulate the calcium dynamics, it has a vital role in mediating the opening of mPTP and the damage in I/R. The CypD-GRP75-IP3R-VDAC complex inhibition improved hypoxia/reoxygenation injury in cardiomyocytes [147]. The hypoxia/reoxygenation process induced the increased interaction of CypD-GRP75-IP3R-VDAC complex and GSK3*β*, leading to cell death. Moreover, GSK3*β* inhibition induces cytosolic and mitochondrial calcium overload, accompanied by reduced cell death and infarct size in reperfused hearts [148]. Due to the negative involvement of SERCA activity in cytosolic Ca^2+^, mitochondrial ROS overproduction, and activation of mitochondrial fission pathway in the myocardium, proteins that could modulate SERCA activity are associated with the susceptibility of the heart to I/R, such as TMX-1 and FUNDC1 [57]. Inhibition of SERCA2b activity in normal conditions by TMX1, a redox-sensitive oxidoreductase localized at MAMs, triggers enhanced ER-mitochondria contacts, thus preventing calnexin during homeostatic conditions.

Diabetic cardiomyopathy (DCM) is characterized by lipid accumulation in the cardiomyocytes and hypertrophy in the left ventricle. DCM is triggered by apoptosis and excessive ROS production, accompanied by the compensatory signaling pathway activation. Cardiac-specific PERK ablation prevents apoptosis in response to high glucose concentrations, which indicates the potential beneficial role of MAM in the prevention of DCM [59]. FUNDC1 contributes to mitochondrial dysfunction after Ca^2+^ increase in diabetes [32]. Additionally, AMPK aberration results in DCM, along with a deregulated number of FUNDC1-related MAMs in diabetic hearts due to the interaction between AMPK and MAMs in cardiomyocytes. Therefore, FUNDC1-related MAM modulation provides new insights into the treatment of DCM.

Heart failure (HF) is a progressive disorder of myocardial remodeling. The increased distance between the SR and mitochondria and diminished mitochondrial Ca^2+^ reuptake are the main characteristics of prohypertrophy induced by norepinephrine in cardiomyocytes [149]. The reduction of Ero1 activity sensitizes hearts to adrenalin, and prevents HF progression in response to hemodynamic overload *in vivo* [149]. Patients diagnosed with HF exhibit a reduced expression of FUNDC1 accompanied by a lower number of SR-mitochondria contacts [127]. FUNDC1 reduction in MAMs induces heart disease. Sig-1R is one of the fundamental proteins of MAMs in HF. Sig-1R inhibition promotes autophagy in cardiomyocytes under oxidative stress conditions [34]. Highly specific agonists of Sig-1R could modulate cardiomyocytes' contractility. In addition, Sig-1R activation represses hypertrophy and cardiomyocyte injury induced by angiotensin II. Sig-1R KO mouse displayed dysfunction and abnormal morphology of mitochondrial as well as cardiac remodeling, resulting in contractile dysfunction. Recently, the interaction of desmin, VDAC, Mic-60 (a central component in mitochondrial contact sites of the cristae organizing system, MICOS), and ATP synthase were identified in heart failure mice, implying the potential beneficial function of such complex in cardiac function.

## 11. Conclusion

In this study, we discussed the molecular structure of MAM and summarized its cellular functions. As a bridge between the endoplasmic reticulum and mitochondria, MAM is mainly involved in calcium homeostasis, lipid metabolism, mitochondrial dynamics, autophagy, apoptosis, and inflammation. Dysregulation of this process has been associated with the pathogenesis of CVD. These data further confirmed the potential use of MAM and related proteins as targets for CVD treatment.

It is important to understand the regulatory mechanisms and targeting proteins involved in the maintenance of MAMs' integrity to explore novel therapeutic targets for the prevention or treatment of heart-associated pathologies. For instance, as fluvoxamine has a high affinity towards Sig-1R, it has been used and tested for the treatment of heart failure and cardiac dysfunction in transverse aortic constriction models in both mice and rats [150]. Additionally, as an antioxidant, N-acetylcysteine was used to treat mitochondrial damage and muscle dysfunction, suggesting the involvement of ROS [151]. Chemical chaperones like 4-phenylbutyrate (PBA) and tauroursodeoxycholic acid can prevent and reverse the established pulmonary arterial hypertension in two rodent disease models by inhibiting the disruption of the ER-mitochondria unit and reducing SR stress [151].

New and more effective therapies are urgently needed for CVD. MAM-related proteins and their inhibitors can directly affect the pathogenesis of CVD, which is of great significance for improving the survival of patients. Future studies should focus on screening new MAM-related protein inhibitors and exploring the possibility and potential of these inhibitors in the treatment of CVD from the molecular mechanism.

## Figures and Tables

**Figure 5:**
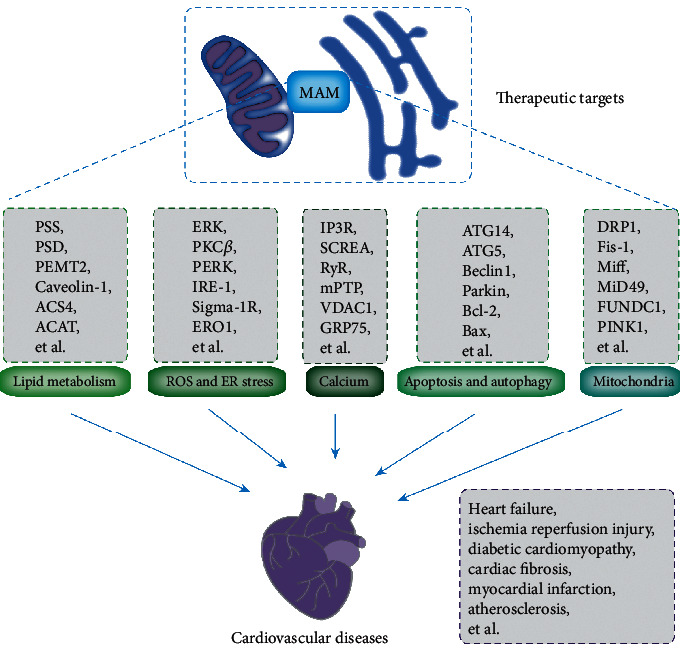
MAM-enriched proteins as potential new therapeutic targets for treatment of CVD-associated pathologies. MAM regulates some major cellular processes, including lipid metabolism, ROS, endoplasmic reticulum pressure, calcium homeostasis, apoptosis and autophagy, and mitochondrial function. The abnormality of these processes often leads to CVD. Notably, the key regulatory proteins of these processes can serve as potential therapeutic targets for CVD.

**Table 1:** Components of MAMs involved in cardiovascular disease.

Proteins	Relevant function(s) in MAMs	Functions in CVD	Expression in cardiovascular system
Protethering proteins			
GRP75	Increased MAM formation and mitochondria Ca^2+^ uptake	Mitochondrial calcium overload and hypoxia/reoxygenation injury in cardiomyocytes	High
IP3Rs	Interacts with GRP75 and VDACs, modulates calcium in MAMs	Upregulation in cardiac hypertrophy. Modulates excitation-contraction coupling in ventricular and atrial cardiomyocytes	Low
VDACs	Interacts with GRP75 and IP3Rs, regulates intracellular Ca^2+^ level	Marked elevation of VDAC1 in myocardial infarction. VDAC1 inhibition alleviates excessive fibrosis in the atrial myocardium	Medium
MFN2	Modulator of ER-mitochondria tethering and mitochondrial fusion	Downregulation in cardiac hypertrophy. MFN2 upregulation ameliorated the cardiac hypertrophy.	Medium
MFN1	Tethering mitochondria to MAMs via interaction with ER-resident MFN2	Represses cardiac hypertrophy and ischemia/reperfusion injury	Not detected
Fis1	Modulates ER-mitochondria tethering and induces apoptosis. Induces mitophagy	Inhibition of the CREB/Fis1 pathway leads to heart disease	High
BECN1	Enhances MAM formation and autophagosomes	Deregulation leads to heart diseases, through altered myocardial autophagy and apoptosis	Low
FUNDC1	Promotes mitochondrial fission and mitophagy. Increases Ca^2+^	Required for cardiac ischemia/reperfusion injury-activated mitophagy	Medium
Parkin	Mediates mitophagy. Increases the ER-mitochondria contacts and induces Ca^2+^ transfer and ATP synthesis	Upregulated during I/R injury	Low
IP3Rs/GRP75/VDAC complex-modulated proteins			
Sig-1R	Prolongs Ca^2+^ signaling; Sig-1R increase represses ER stress response, whereas Sig-1R decrease induces apoptosis	Sig-1R activation represses hypertrophy and cardiomyocyte injury. Sig-1R KO displays cardiac remodeling	High
CypD	Regulates Ca^2+^ transfer from the ER to mitochondria through IP3R1	The CypD/GRP75/IP3R/VDAC complex inhibition improved hypoxia/reoxygenation injury in cardiomyocytes	NA
GSK3*β*	Inhibition of GSK3*β* results in decreased ER Ca^2+^ release as well as sensitivity to apoptosis	GSK3*β* inhibition reduced infarct size in reperfused hearts	Not detected
Antitethering proteins			
CAV1	Negatively regulates the formation of MAMs and impairs Ca^2+^ transfer	CAV1 ablation aggravates cardiac dysfunction and decreases survival in myocardial ischemia	Medium
Upstream regulators of the formation of MAMs			
p38 MAPK	Phosphorylation of Gp78 at S538 by p38 MAPK inhibits MAM formation and mitochondrial fusion by promoting degradation of MFN1/2	p38 MAPK has been implicated in cardiomyocyte dysfunction and apoptosis	Medium
FOXO1	Augments MAM formation by inducing PDK4 and promotes mitochondrial Ca^2+^ accumulation, mitochondrial dysfunction, and ER stress	FOXO1 protein is associated with ischemic heart disease (IHD)	Not detected

## Data Availability

All data generated or analyzed in this study are available from the corresponding author on reasonable request.
